# Numerical and Experimental Analysis of Titanium Sheet Forming for Medical Instrument Parts

**DOI:** 10.3390/ma15051735

**Published:** 2022-02-25

**Authors:** Wojciech Więckowski, Maciej Motyka, Janina Adamus, Piotr Lacki, Marcin Dyner

**Affiliations:** 1 Faculty of Mechanical Engineering and Computer Science, Czestochowa University of Technology, 69 Dabrowskiego St., 42-201 Czestochowa, Poland; wojciech.wieckowski@pcz.pl; 2 Faculty of Mechanical Engineering and Aeronautics, Rzeszow University of Technology, 12 Powstancow Warszawy Ave., 35-959 Rzeszow, Poland; 3 Faculty of Civil Engineering, Czestochowa University of Technology, 69 Dabrowskiego St., 42-201 Czestochowa, Poland; janina.adamus@gmail.com (J.A.); piotr@lacki.com.pl (P.L.); 4 Faculty of Science and Technology, Jan Dlugosz University in Czestochowa, 13/15 Armii Krajowej Ave., 42-200 Czestochowa, Poland; m.dyner@ujd.edu.pl

**Keywords:** sheet titanium forming, Grade 2 titanium, environmentally benign lubricant, numerical analysis, medical instruments

## Abstract

The paper analyses the forming of the surgical instrument handles made of Grade 2 titanium sheets. Sheet metal forming is a technology ensuring high strength and light weight of products. Replacing stainless steels with titanium further reduces instrument weight and additionally provides the required resistance to corrosive environments typical for surgeries. The low instrument weight is important to prevent fatigue of surgeons and allow them to maintain high operational accuracy during long term surgeries. The numerical analysis of the technological process was performed in order to adapt it to forming tool handles using titanium sheets instead of steel sheets. The numerical calculations were experimentally verified. It was found that, in the case of titanium handles, it is necessary to use a blank holder in the first forming operation to eliminate sheet wrinkling in the flange area. The shape and dimensional accuracy of the drawn part after trimming were high enough and the 4th forming operation became unnecessary. Moreover, the process modification included lubrication using rapeseed oil with the addition of boric acid, which effectively prevents the galling of titanium on the working surfaces of the steel tools and ensures a more uniform distribution of plastic strains in the drawn part.

## 1. Introduction

Medical instruments are mostly made of highly-alloyed stainless steels, including martensitic, ferritic and austenitic steels. Only in justified cases, materials such as platinum, titanium, tantalum and their alloys are used [1]. In the case of gripping parts, used for holding instruments in the operator’s hand, aluminium alloys and, recently, plastics, as well as composite materials are also used. In order to increase their corrosion resistance, as well as antibacterial and antiviral properties, instruments are subjected to surface treatment [2]. In [3], Xue et al. summarised the latest advances in surface modification techniques, especially titanium and its alloys, for biomedical applications. The surface techniques include plasma spray, physical vapor deposition, sol-gel, micro-arc oxidation, etc. The conditions in which medical instruments are used, namely long-term contact with organic tissue, body fluids and disinfectants are the reasons why the materials used for medical instruments should have good corrosion resistance. As emphasised by Gherlone et al. [4], in the light of the COVID19 pandemic, when CoV-2019 can be transmitted not only directly from person to person by respiratory drops, but also by direct contact with contaminated material, it is important that medical instruments were made of materials resistant to disinfectants. Frequent sterilisation accelerates corrosive processes and induces corrosive wear of the tools.

The second very important factor taken into account when choosing materials for medical instruments is their weight. The light weight of surgical instruments is of particular importance during long-term surgical operations, often requiring high precision. In addition, parts of medical instruments, such as the working and gripping parts and connectors, must exhibit appropriate mechanical properties ensuring the transfer of loads occurring during the work of instruments, without changing the functional characteristics of individual parts of the instruments. The normative recommendations relating to materials used for medical instruments are described in [5,6].

The use of gripping parts made of thin-walled elements is a solution that allows the weight of medical instruments to be reduced. Among the various manufacturing techniques such as machining, casting or 3D printing, metal forming provides the greatest strength to thin-walled elements [7]. Products manufactured by sheet metal forming, despite the small thickness of the walls of the drawn part, are characterised by high strength due to strain hardening taking place during cold working. In work [8,9], discussing the latest trends in metal working, sheet metal forming is indicated as a process enabling the production of light and durable products, so desired in many areas of life, including the medical industry. To advance the rehabilitation efficacy and shorten the recovery period of patients, Cheng et al. [10] proposes incremental sheet titanium forming for the production of biomedical implants. However, this flexible forming technique still requires a great deal of effort to increase the geometric accuracy to make the drawn part fit properly. Scratches and marks, caused by the forming tool, pose problems which are difficult to overcome, so the process is still in the experimental phase.

An additional reduction in the mass of drawn parts, while maintaining appropriate strength, can be obtained by using sheets of light and high-strength materials such as titanium and its alloys. Up to 600 °C, titanium alloys have the highest strength-to-weight ratio among all construction metals [11]. Biocompatibility is an additional advantage of titanium regarding medical applications, especially due to postoperative complications in the case of using implants and surgical instruments made of materials sensitising patients, which the authors of article [12] pay attention to. According to [13,14], titanium does not cause an undesirable biological interaction in contact with living tissue. The beginnings of using titanium and its alloys for structural elements date back to the end of the 1940s. With the development of implants in the 1960s, the first surgical titanium instruments also appeared [15]. Somewhat later, in the 1980s, titanium started to be used to reduce the weight of car parts [16]. However, due to the high cost of producing and processing titanium materials, their use in civil applications is still limited, mainly to produce implants and high-end sports equipment, as well as in the automotive industry for high-end cars and racing ones. Furthermore, although at the beginning of the 1980s titanium sheets began to enter the construction market mainly as roofing materials, curtain walls and ventilation shafts [17,18], the aviation industry is still the largest customer of titanium products. Unfortunately, at ambient an temperature, sheets made of materials with high specific strength, such as titanium alloys, are characterised by low formability. Among titanium materials, only Grade 1 and Grade 2 commercially pure titanium sheets can be relatively easily formed in ambient temperatures with typical dies used for sheet steel forming [11,19]. Other commercially pure titanium (Grade 3 and 4) as well as titanium alloys belong to hard-to deform materials in ambient temperatures. To solve this problem, hot forming is applied. Wang et al. [20] propose Fast light Alloys Stamping Technology (FAST), where fast heating is employed to maintain the post-form strength but not impairing the formability. Moreover, Tian and Li [21] studied the hot stamping process for forming complex-shaped titanium alloy components, combining heat treatment and fast stamping. Although hot forming improves the drawability, it requires more expenditure on heating and the use of protective atmospheres to prevent increased gas (oxygen, hydrogen and nitrogen) absorption by titanium.

The literature review shows that works in the field of cold forming titanium and its alloys, especially sheet metal forming, are almost unique materials, what is also emphasised in the work [22] from 2021. Scientific works mainly concern the study of titanium properties and microstructure, heat and surface treatment, as well as corrosion problems. These publications mainly emphasise the advantages of titanium and its alloys compared to steel, especially the favourable mechanical strength in relation to titanium density, high resistance to most common corrosive environments and good biocompatibility. Usually, the research concerns the drawn part with simple geometry, such as a spherical cup. There is no detailed information on the process parameters of cold forming titanium sheets. In [22], plastic behaviour and formability of the commercially pure titanium CP Grade 2 hcp-titanium T40 was examined. The Authors paid attention to the significant influence of sheet anisotropy on the occurrence of instability and distribution of plastic strains. Chinapareddygari et al. [23] assessed the stretchability of Grade 4 commercially pure titanium sheets on the basis of tensile tests (yield strength, ultimate tensile strength, elongation), the plastic strain ratio (*r*-value) and forming limit diagram (FLD). Lin et al. [24], when analysing the deep drawing behaviour of commercially pure titanium at room temperature, pointed out that earing is a result of planar anisotropy and tension-compression asymmetry (TCA). TCA reduced the thickness of the deep drawn sections, increased the earing ratio, and influenced the drawing force. The earing profiles depended strongly on the texture evolution. Pham [25,26] examined both experimentally and numerically the anisotropic yielding behaviour and the distortional hardening behaviour of commercially pure titanium sheets during the hydraulic bulge test. Chen and Chiu [27] stated that the low ductility of commercially pure titanium at room temperature results from its hexagonal close-packed crystal structures, and thermal activation is required to increase its ductility and formability. Mohanraj and Elangovan [28] also showed that the formability of Grade 2 titanium sheets can be improved at elevated temperatures. However, the manufacturing process at room temperature is always preferred for the reason of cost-effectiveness. Therefore, scientists are constantly looking for new methods of the cold forming of sheets of hard-to-deform materials. Adamus and Lacki [29,30] indicate the possibility of improving the drawability of titanium sheets thanks to the use of semi-flexible tools. Aydogan et al. [31] used hydroforming with a membrane diaphragm to shape Grade 2 commercially pure titanium. They pointed out that hydroforming reduces the springback value of the sheets and gives a more uniform strain distribution comparing to other forming methods due to the smooth action of fluid pressure. However, hydroforming is an inefficient and very demanding process. It requires high-capacity presses, a qualified workforce, etc. Kumar [32] underlined that material formability depends on intrinsic material properties and prevailing process parameters, especially lubricating conditions. Kakulite [33] added that galling is one of the major disadvantages of friction, which is commonly observed in sheet metal forming, and that the use of suitable lubricants and coatings can be two of the easiest, most economical and efficient methods to mitigate galling. Adamus and Dyja [34] achieved good results in decreasing the coefficient of friction during sheet-titanium forming thanks to the application of lubricants based on vegetable oils with an additive of boric acid. In [35], the authors concluded that anti-adhesive coatings put on the working parts of the dies are additional protection against galling when the lubricant film breaks under the high unit pressure occurring in the sheet-titanium forming process. The authors’ experience to date has shown that tool geometry, especially the die and punch fillet radii and the blank holder force are also key factors in sheet metal forming.

In this paper, an analysis of the forming process of the gripping part of a surgical instrument previously made of steel 1.4021 [36] was carried out. Grade 2, the most commonly used commercially pure titanium in technology and medicine, was selected as the material for handle production. This choice was made due to its biocompatibility, quite good formability resulting from the ratio of yield point to tensile strength of about 0.7 and elongation of about 20%, low density of 4500 kg/m^3^, almost half that of steel, high corrosion resistance, and good weldability. As Grade 2 titanium belongs to α alloys which undergo strain hardening sheet thinning occurring during plastic forming, and will be compensated by material hardening. Due to the fact that the drawn part has an elongated rather complex shape, it is important to take into account the sheet plastic anisotropy and the appropriate location of the blank in relation to the rolling direction.

Theoretical analyses of the sheet metal forming processes were carried out with the commercial PAMStamp 2G [37] programme, dedicated to sheet metal forming processes. The results of the numerical analyses were experimentally verified.

The novelty of the work refers to the use of grade 2 titanium on parts usually made of steel, which allows for a weight reduction of about 47%, and the use of technological lubricant based on vegetable oil with an additive of boric acid, which effectively lowered the frictional resistance during forming from 0.4 in dry conditions to 0.1 with lubrication. This lubricant completely eliminates the galling phenomenon, which allows for forming titanium handles with the use of rigid steel tools. What is very important also, is that the lubricant does not pose a threat to the environment. An additional advantage of titanium products is the possibility of colouring them by performing anode oxidisation to the titanium, thanks to which it is easy to distinguish tools during a surgical procedure.

## 2. Materials and Methods

### 2.1. Aim and Scope of Work

**Figure 1:**
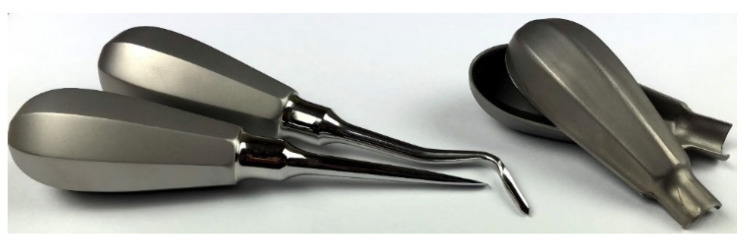
View of handle parts of surgical instruments.

In the work, the cold forming of the components of a surgical instrument handle, which are presented in Figure 1, is analysed. The main goal of the study is to modify the technological process, which is currently used for forming steel handles, in order to make it possible to produce them from titanium sheets.

**Figure 2:**
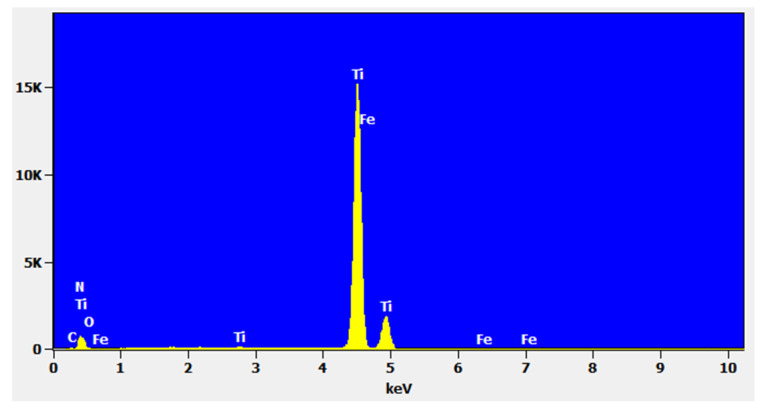
EDS spectrum of Grade 2 CP titanium.

The handle consists of two parts formed from sheet metal, which are then joined by welding. In order to reduce the instrument weight, it was proposed to make these parts from Grade 2 commercially pure (CP) titanium sheet (Table 1), produced by Kobe Steel, LTD, Kakogawa, Japan according to EN 10204 -Material Certificate 3.1 [38]. Its chemical composition was confirmed using energy-dispersive X-ray spectroscopy (EDS)—Ti and Fe—99.8 and <0.1 wt.%, respectively (Figure 2). Until now, these parts were made of 1.4021 steel sheet (Stahl Krebs, Solingen, Germany).

The analyses include:

− experimental forming of the drawn parts in industrial conditions;
− numerical simulations of the sheet metal forming process, using the PAMStamp 2G programme, based on the finite element method (FEM).

Mechanical properties (Young’s modulus *E*, yield stress *R_p_*_0.2_, tensile strength *R_m_*) and technological ones (Lankford coefficient *r*, strength coefficient *K* and strain hardening exponent *n*) of the analysed sheet were determined in the static uniaxial tensile test in accordance with [39]. The tests were carried out on Zwick Z50 (Zwick Roell Polska Sp.zoo. Sp.k., Wroclaw, Poland) tensile testing machine. To determine forming limit diagram (FLD) of the sheet, ARAMIS 5M System was used. It is a non-contact measuring system based on digital image correlation and photogrammetry. To determine coefficient of friction, a strip drawing test was applied. Strip drawing test is used to model friction condition in sheet metal forming, i.e., between the punch/die and deformed sheet.

### 2.2. Experimental Tests

**Figure 3:**
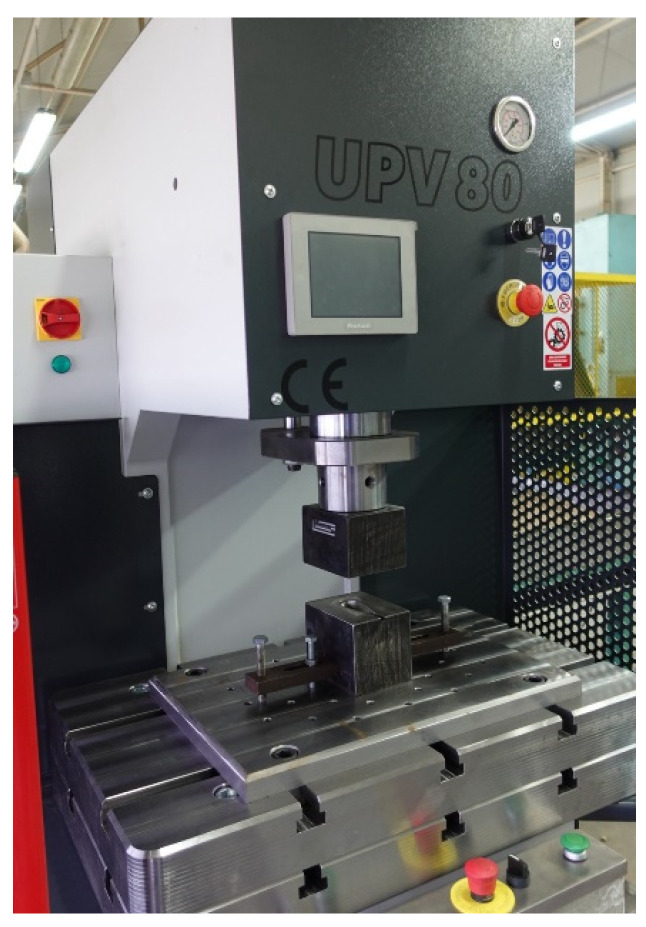
View of forming tool installed in UPV80 hydraulic press.

Tests of sheet metal forming were performed on typical steel rigid tools used for the production of steel sheet handles. The tool was mounted in UPV80 hydraulic press (Figure 3) with pressure of 80 T, which is used in the Factory of Medical Instruments CHIRMED, Rudniki, Poland for mass production of different drawn parts. The manufacturing process of the handle part to date used in the factory consists of four operations: initial and supplementary forming, trimming the flange and final forming. All the forming operations are performed in a tool consisting of a die and a punch. Rectangular Grade 2 titanium sheet blanks with dimensions of 85 mm × 57 mm × 1 mm were used to form the drawn parts. The forming speed was 5 mm/s. To reduce the forming resistance and to prevent titanium sticking to the working surface of the steel tools in the subsequent forming operations, a vegetable oil-based lubricant with the addition of boric acid was used. According to [34], using this lubricant when forming titanium sheets significantly reduces the coefficient of friction between the deformed material and the working surface of the tool from 0.4 in the absence of lubrication to 0.1 in the presence of the lubricant.

### 2.3. Numerical Model

**Figure 4:**
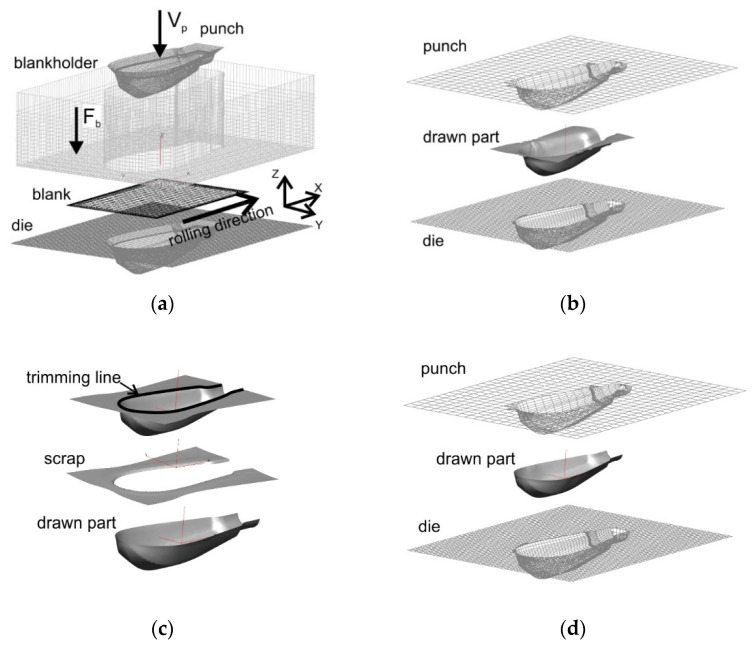
Numerical model of forming handle part (with mesh of finite elements): (**a**) initial forming; (**b**) supplementary forming; (**c**) flange trimming; (**d**) final forming.

Numerical models of the individual forming operations were developed on the basis of the actual geometry of the forming tools. In the calculations, the process parameters used in the experimental tests were assumed. The Solidworks System was used to create the tool geometry. Then the geometry was exported to the PamStamp 2G System. The working surfaces of the tools and the sheet metal surface were meshed with four-node shell elements (see Figure 4).

In the case of the initial forming operation, two variants of numerical simulations were analysed:

1. forming without a blank holder;
2. forming with a blank holder.

In the numerical calculations, the friction and lubrication conditions were described by the coefficient of friction. In the case of forming without the blank holder, the coefficient of friction μ = 0.1 was assumed on the contact surfaces between the die and the deformed sheet to reflect the lubrication conditions prevailing during tests in industrial conditions. In the case of forming with the blank holder, two different coefficients of friction were assumed:

- μ = 0.1 for lubricated contact surfaces, i.e., on the contact “die-deformed material-blank holder”;
- μ = 0.4 for unlubricated contact surfaces.

In the analysed numerical models, the following boundary conditions were assumed: the dies were deprived of all degrees of freedom, while the punch and the blank holder could move in the Z direction. Velocity vector *Vp* was assigned to the punch, while force vector *Fb* was applied to the blank holder. The blank sheet had all degrees of freedom. The rolling direction of the sheet was assumed in accordance with the *X* axis parallel to the longer edge of the blank.

In the numerical calculations, an elastic-plastic Hill model for the deformed material with nonlinear strain hardening described by Hollomon’s law was adopted:

(1) $$\sigma_{p}=K\varepsilon^{n},$$

where: *K*—strength coefficient, *n*—strain hardening exponent.

The basic strength parameters, including the strength coefficient and strain hardening exponent of the Grade 2 titanium sheet, were determined in the static uniaxial tensile test in accordance with the PN-EN ISO 6892-1 standard. Normal anisotropy ratios *r*_α_ were also determined experimentally in the tensile test of sheet metal strips cut along (0°), transversely (90°) and at an angle of 45° in relation to the rolling direction of the sheet, in accordance with [40]. The material data for the Grade 2 sheet, which were assumed in the calculations, are given in Table 2.

**Figure 5:**
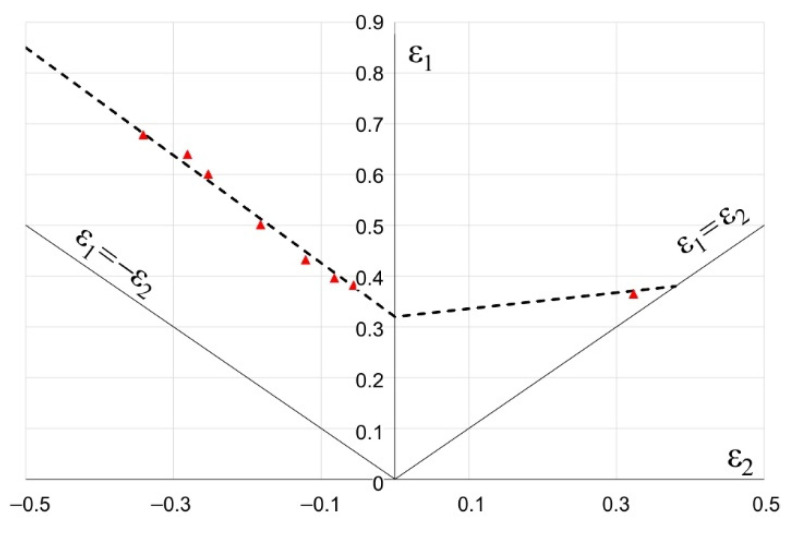
Forming limit diagram for Grade 2 titanium sheet, *t* = 1.0 mm.

The forming limit diagram (FLD) of a Grade 2 titanium sheet with thickness *t* = 1 mm, which is presented in Figure 5, was determined experimentally according to the methodology described in [41].

## 3. Results

### 3.1. Experimental Test Results

**Figure 6:**
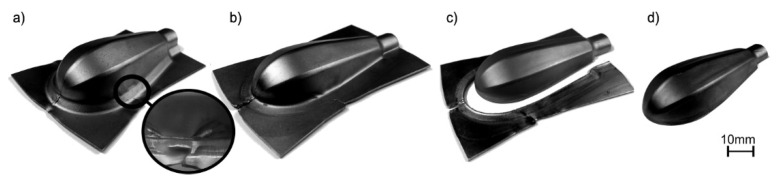
Subsequent stages of forming handle part from Grade 2 titanium sheet: (**a**) initial forming with magnification of wrinkling area; (**b**) supplementary forming; (**c**) trimming; (**d**) final forming.

The results of the forming process are presented in Figure 6.

**Figure 7:**
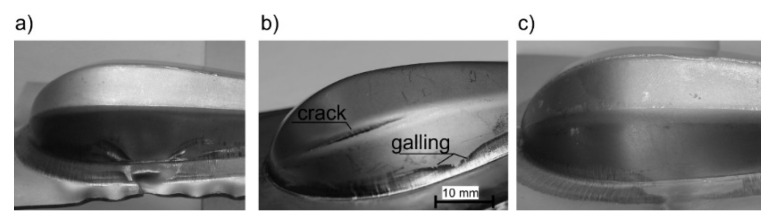
Results of forming: (**a**) with no blank holder—visible wrinkling, (**b**) with blank holder in dry conditions—visible crack and marks of galling, (**c**) with blank holder and lubrication—there are no defects.

The experimental tests showed that it is possible to produce the handle parts of Grade 2 titanium sheet with standard steel tools. However, the lack of a blank holder in the initial forming process results in the wrinkling of the sheet, both in the flange of the drawn part and on its surface (Figure 7a). On the other hand, forming with the blank holder in dry conditions very often leads to fracture (Figure 7b). Moreover, the experiments showed a high tendency to create titanium protrusions on the working surfaces of the steel die, which in turn leads to galling (Figure 7b) and deterioration of the quality of the drawn part surface. Titanium protrusions cause scratches and dents that cannot be removed in subsequent forming operations. Therefore, lubrication was necessary to separate the rubbing surfaces (i.e., the deformed material—titanium—and the die). Lubricant based on rapeseed oil with an additive of boric acid [42] was used. The use of the blank holder and technological lubricant, made it possible to obtain a good-quality titanium drawn part (Figure 7c).

To eliminate this defect, it was decided to modify the forming process using numerical simulations.

### 3.2. Numerical Calculations Results-Discussion

**Figure 8:**
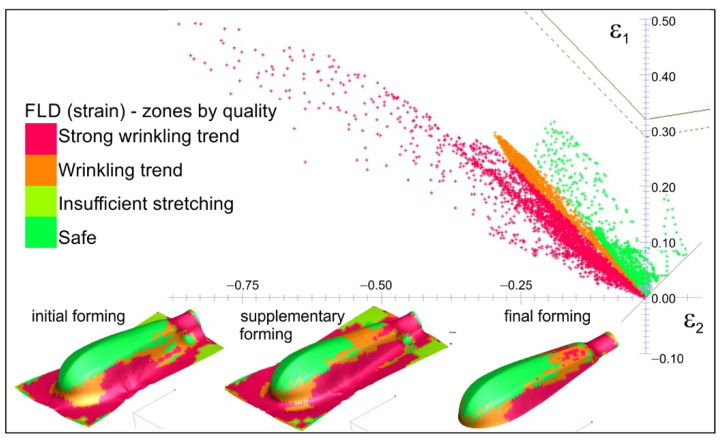
Strain distribution (zones by quality) in comparison with forming limit diagram.

The results of the numerical calculations in the form of plastic strain distribution, as well as thickness distribution in the drawn parts for all the stages of forming the titanium handle are presented. Additionally, the distribution of quality zones in comparison with the FLD of the deformed sheet are given. The simulation results of forming the handle parts in conditions corresponding to the experimental tests, i.e., without a blank holder during initial forming and with lubrication of the die surface, which are presented in Figure 8, are in good agreement with the experimental tests (also see Figure 6).

**Figure 9:**
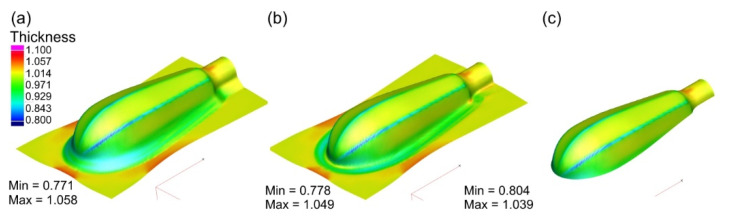
Thickness distribution in drawn part wall after: (**a**) initial forming; (**b**) supplementary forming; (**c**) final forming in dry conditions.

**Figure 10:**
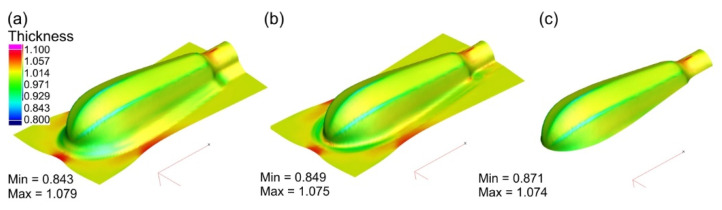
Thickness distribution in drawn part wall after: (**a**) initial forming; (**b**) supplementary forming; (**c**) final forming performed with lubricant.

**Figure 11:**
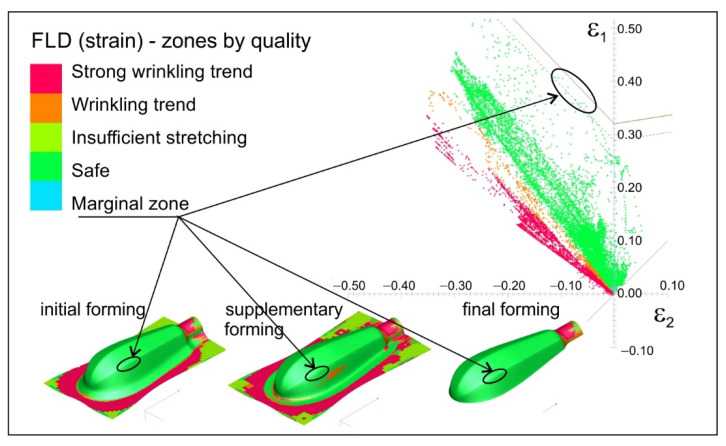
Plastic strain distribution in comparison with forming limit diagram (variant: forming with blank holder and without lubrication).

**Figure 12:**
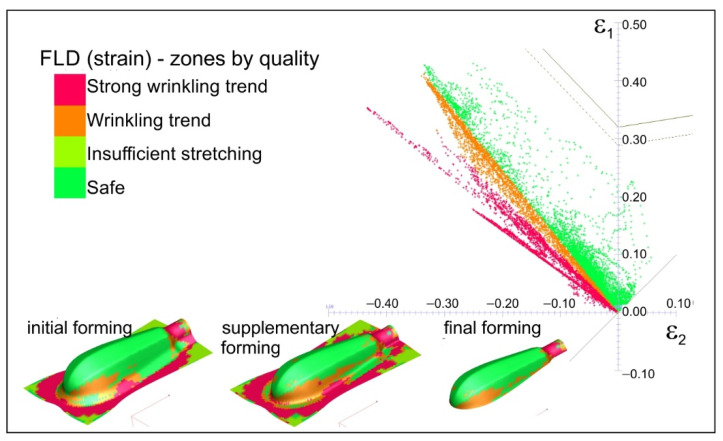
Plastic strain distribution in comparison with forming limit diagram (variant: forming with blank holder and with lubrication).

During the initial forming operation, the areas with a strong tendency to wrinkle are visible in the flange as well as on the drawn part surface. This tendency also continues during subsequent forming operations. The use of the blank holder, as demonstrated by the numerical simulations, eliminated the wrinkling phenomenon. Simulations were carried out under various friction conditions between the sheet material and the working surfaces of the tool, i.e., with and without lubrication. The results are presented in Figure 9, Figure 10, Figure 11 and Figure 12. The thickness distributions in the drawn parts after the initial, supplementary and final forming operations are presented in Figure 9 and Figure 10, respectively, for forming in dry conditions and with lubrication.

Greater thinning of the drawn part walls is observed in the case of forming in dry conditions. The greatest thinning occurs on the edges of the handle, in the area with the small fillet radius. The application of lubricant (μ = 0.1) causes a more uniform thickness distribution—the wall thinning is smaller.

During forming without lubrication, significant material deformation along the edge of the drawn part occurs, which results in excessive thinning of the sheet and can lead to its fracture. This is reflected in the points in the marginal zone just below the forming limit curve (Figure 11). In this area, cracking occurred during the actual forming process (see Figure 7b).

Introducing lubricant between the rubbing surfaces decreases the frictional resistance. All the points representing strain values are below the FLD in the safe zone (see Figure 12). This means that the risk of cracking is eliminated.

**Figure 13:**
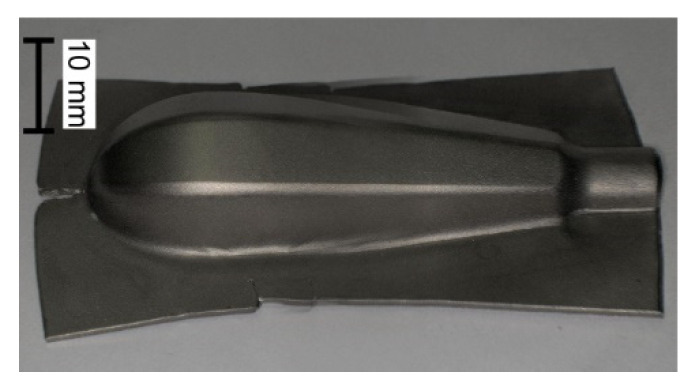
View of drawn part formed in presence of lubricant and blank holder.

On the other hand, the tendency to wrinkling grows because lubrication decreases frictional resistance in the contact zone: “die-deformed material–blank holder” makes material flow to be easier. In the actual forming process, when both lubrication and the blank holder were used to tend to, wrinkling was not observed. Moreover, the lubricant prevented the sticking of the deformed material (titanium) to the forming tools, as evidenced by the smooth surface of the drawn part shown in Figure 13.

**Figure 14:**
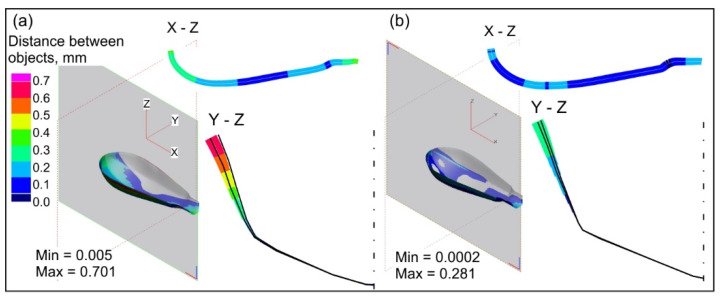
Springback after flange trimming: (**a**) initial forming without blank holder, (**b**) initial forming with blank holder and lubrication.

Moreover, the numerical analyses show that the forming parameters, such as the blank holder force and lubrication, have an impact on the dimensional accuracy of the obtained parts. The values of springback occurring after flange trimming are shown in Figure 14.

The highest springback is observed after trimming the flange in the case of forming without the blank holder (Figure 14a). The maximal distance between the nominal and formed drawn part is about 0.7 mm. Applying the blank holder force of 20 kN reduced this distance to about 0.3 mm. No effect of lubrication on springback was observed. If the forming process is performed with the blank holder, the forth operation, i.e., final forming, increases the shape and dimensional accuracy of the drawn part only slightly. Thus, if it were decided to modernise the process and introduce the blank holder in the initial forming operation, then final forming after trimming can be omitted. The use of the blank holder is advisable in view of eliminating sheet wrinkling, which makes it difficult to further form the handle parts of the instrument.

### 3.3. Experimental Verification of Numerical Results

**Figure 15:**
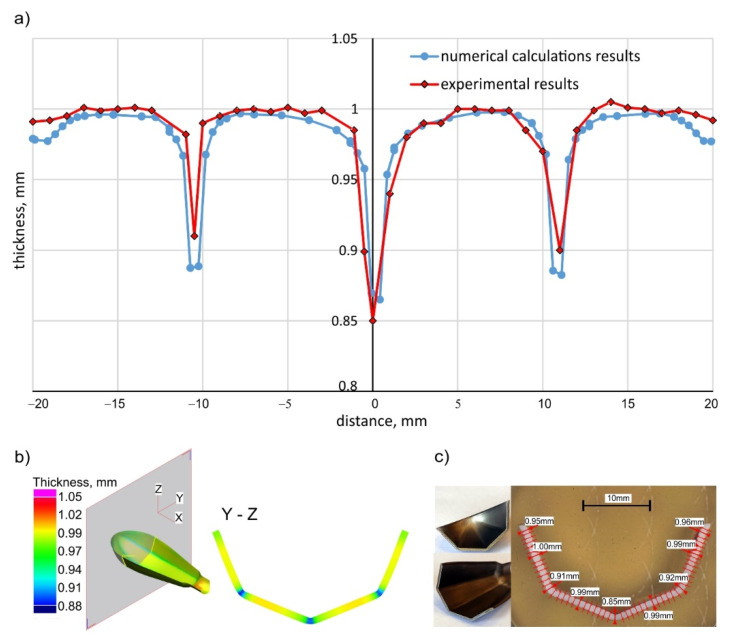
Thickness distribution: (**a**) comparison of experimental and numerical results; (**b**) numerical calculation results; (**c**) experimental verification.

The results of the numerical calculations, namely the wall thicknesses of the drawn part, were compared with thicknesses of the actual drawn part (Figure 15). For this purpose, the final drawn part was cut with a wire EDM machine, and then the wall thicknesses were measured at the selected points (along the wall of the drawn part every 1 mm—Figure 15c) using a light microscope.

Modification of the technological process, consisting in the use of blank holder force in the first operation (*Fb* = 20 kN) and lubrication with rapeseed oil with an additive of boric acid (coefficient of friction μ = 0.1), resulted in obtaining a titanium drawn part with the required shape and dimensions. The maximal difference in thickness of the drawn part walls between the numerical and experimental results is about 2%. Moreover, there was no tendency of the sheet to wrinkle or scratches on the outer surface of the drawn part (see the forming results in Figure 13). Additionally, forming with the proposed lubrication protects the working surfaces of the tool against the galling of the titanium.

## 4. Conclusions

On the basis of the experimental and numerical studies the following conclusions can be drawn:

1. There is a possibility of forming shallow drawn parts, such as the analysed handle of surgical instruments, of Grade 2 CP titanium sheets using standard steel tools, usually used for forming steel drawn parts.
2. The use of Grade 2 CP titanium sheet reduces the handle weight of by about 47%, which significantly improves the comfort of using the tools during surgery.
3. The blank holder force has a dominant influence on the dimensional accuracy of the drawn part; according to the numerical calculations, the blank holder force equals 20 kN.
4. Although the numerical calculations show a small effect of the lubrication on the dimensional accuracy of the drawn parts, the experimental tests demonstrate that lubrication must be used to avoid galling.
5. The blank holder force and lubrication synergy result in a more uniform plastic strain distribution (compare Figure 11 and Figure 12), thus decreasing the risk of fracture of the drawn part.
6. The use of lubricant facilitates flow of the deformed material and improves smoothness of the drawn part surface, which improves the quality of the product.
7. The lubricant, applied to the sheet surface according to patented method [42], is environmentally friendly and has no harmful effect on people

## Figures and Tables

**Table 1:** Nominal chemical composition of Grade 2 CP titanium.

Element	Ti	Fe	O	C	N	H
Content, wt.%	≥98.9	≤0.30	≤0.25	≤0.09	≤0.03	≤0.015

**Table 2:** Material data for Grade 2 commercially pure titanium sheet *t* = 1 mm.

Young’s modulus *E*, GPa		105.00
Yield stress *R_p_*_0.2_, MPa		354.30
Tensile strength *R_m_*, MPa		472.40
Poisson’s ratio *ν*, -		0.34
Density *ρ*, g/cm^3^		4.50
Lankford coefficient	*r*_0_, -	2.49
	*r*_45_, -	4.50
	*r*_90,_ -	5.20
Strength coefficient *K*, MPa		724.40
Strain hardening exponent *n*, -		0.144

## Data Availability

The data presented in this study are available on request from the corresponding author.
